# Effects of cardiac rehabilitation on elderly patients with Chronic heart failure: A meta-analysis and systematic review

**DOI:** 10.1371/journal.pone.0273251

**Published:** 2022-08-25

**Authors:** Zhuang Chen, Ming Li, Chenghua Yin, Youbo Fang, Ye Zhu, Jing Feng

**Affiliations:** 1 Department of Cardiovascular Medicine, The Fifth People’s Hospital of Jinan, Jinan, Shandong, China; 2 Department of Emergency, Yidu Central Hospital, Weifang, Shandong, China; University of Dundee, UNITED KINGDOM

## Abstract

**Aims:**

The purpose of this study was to investigate the effects of cardiac rehabilitation (CR) on elderly patients with Chronic heart failure (CHF) by literature search and meta-analysis.

**Methods:**

We conducted an electronic search on PubMed, Cochrane Library, Embase, CNKI, Wanfang, and VIP database platforms. The search period was from the establishment of the database to November 2021 for randomized controlled studies (RCTs) related to the effects of CR on elderly patients with CHF. The RevMan 5.4 was used for meta-analysis.

**Results:**

This study included 16 articles involving a total of 1782 patients, including 892 in the CR group and 890 in the control group. Meta-analysis showed that compared with conventional interventions, CR increased left ventricular ejection fraction in elderly patients with CHF [mean difference (MD):5.73,95% confidence interval (CI):2.05 to 9.40,Z = 3.05,P = 0.002], and decreased left ventricular end-diastolic diameter in elderly patients with CHF (MD:-4.82,95%CI:-7.49 to 15,Z = 3.54,P = 0.0004), increased the 6-minute walk test distance (MD:62.66,95% CI:44.40 to 80.92,Z = 6.72,P<0.00001), decreased the rehospitalization rate (OR:0.32,95%) CI: 0.21 0.49, Z = 5.33, P < 0.000001).

**Conclusions:**

CR can improve cardiac function, prognosis and reduce rehospitalization rate of elderly patients with CHF.

## Introduction

CHF is a chronic, spontaneously progressive disease caused by a variety of etiologies, characterized by dyspnea, fatigue, and fluid retention [1]. Heart failure not only seriously damages the physical and mental health and quality of life of patients, but also brings a heavy economic burden to the family and society [2]. According to epidemiological surveys, the prevalence of heart failure in developed countries is 1.5% to 2.0%, and the prevalence of people aged ≥ 70 years is ≥ 10% [3].With the aging of the population in my country and the expansion of chronic diseases, the prevalence, mortality and rehospitalization rates of heart failure continue to increase [4]. The prevalence of heart failure among Chinese people aged 80 years or older is 12%, and advanced age has become an important factor for poor prognosis in patients with heart failure [5]. The prevalence, mortality and rehospitalization rates of heart failure continue to increase, and advanced age has become an important factor in poor prognosis of heart failure patients. Elderly patients with heart failure have many comorbidities, and their management is specific and complex [6]. CR is a professional prevention and treatment system integrating biomedicine, sports medicine, nutrition medicine, psychosomatic medicine and behavioral medicine. CR currently includes three stages, respectively is I period rehabilitation (nosocomial), II period rehabilitation (outside early rehabilitation hospital) and III period rehabilitation (maintain), IIphase of the CR is also known as outside early rehabilitation hospital or outpatient rehabilitation, in 1 ~ 6 months after hospital discharge, as the core of the recovery phase, both I period of rehabilitation, is also the foundation of III period rehabilitation, CR exercise can induce adaptive changes in the function and structure of cardiovascular, respiratory, musculoskeletal and metabolic systems, and the nature and magnitude of the changes depend on the type, intensity and duration of exercise [7]. CR intervention for CHF has been carried out for many years, and evidence-based medical evidence shows that CR can benefit patients with CHF [8]. CR can improve the cardiac function and structure of patients with heart failure, improve exercise tolerance and quality of life in patients with heart failure, reduce the mortality and readmission rates of patients with heart failure, and control social medical costs [3,9]. At present, there are many studies on the evaluation of the therapeutic effect of CR on patients with CHF, but there is no systematic review article pointing out the impact of CR on the therapeutic effect of elderly patients with CHF. The aim of this meta-analysis was to evaluate the impact of CR on elderly patients with CHF.

## Materials and methods

We followed the Preferred Reporting Items for Systematic Reviews and Meta-Analyses (PRISMA Statement) guidelines to conduct this systematic review and meta-analysis [10].

### Search strategy and research options

We conducted an electronic search on PubMed, Cochrane Library, Embase, CNKI, Wanfang and VIP database platforms. The search period was from the establishment of the database to November 2021 without language restriction. A combination of subject words and free words is used. The following search terms are used for CR: CR OR Cardiac Rehabilitations OR Rehabilitation, Cardiac OR Rehabilitations, Cardiac OR Cardiovascular Rehabilitation OR Cardiovascular Rehabilitations OR Rehabilitation, Cardiovascular OR Rehabilitations, Cardiovascular; The following search terms were used for the elderly: Aged OR Elderly; The following search terms were used for HF: Heart Failure OR Cardiac Failure OR Heart Decompensation Decompensation, Heart OR Heart Failure, Right-Sided OR Heart Failure, Right Sided OR Right-Sided Heart Failure OR Right Sided Heart Failure OR Myocardial Failure OR Congestive Heart Failure OR Heart Failure, Congestive OR Heart Failure, OR Left-Sided OR Heart Failure, Left Sided OR Left-Sided Heart Failure OR Left Sided Heart Failure.

### Inclusion criteria

(1) Literature search time > 2000 years; (2) Intervention measures: CR + other treatments (experimental group), other treatments (control group); (3) Age: elderly patients (≥60 years old); (4) CHF patients; (5) Type of study: randomized controlled trial.

### Exclusion criteria

(1) Original text not available; (2) Retrospective studies or prospective studies or abstracts; (3) Systematic reviews; (4) Non-CHF or other diseases (5) Incomplete outcomes or treatments; (6) Unavailable complete data.

### Data extraction

Two researchers (Z Chen and J Feng) independently conducted research screening and data extraction; If there is any disagreement, resolve it through discussion or negotiation with the third researcher (Y Fang). The data to be extracted in this study include: (1) general information of the study (title, first author, publication year, literature source); (2) Basic characteristics of the study (age, time, sample size, study protocol design, intervention measures, outcome evaluation indicators).

### Evaluation of the quality of evidence

Grading of evidence uses Grading of recommendations assessment, development and evaluation (GRADE) system. Grade the limitations, inconsistencies, inconsistencies, uncertainties and publication bias of evidence, and divide the quality of evidence into 4 grades, namely, high quality, medium quality, low quality and very low quality. To evaluate the outcome index of this study with specific evidence quality.

### Quality assessment

The "Bias Risk Assessment" tool 2.0 recommended by the Cochrane Collaboration (https://www.riskofbias.info/welcome/rob-2-0-tool) to have included in the quality of literature evaluation, It includes the following five modules: bias arising from randomization process, bias of deviating from established intervention, bias of missing outcome data, bias of outcome measurement and bias of selective reporting of results. The bias risk of each module can be divided into three levels of "low risk", "certain risk" or "high risk" according to manual standards.

### Statistical analysis

RevMan 5.4 software was used for statistical analysis of the data. Odds ratio (OR) was used as the effect size, 95% CI represented the outcome, and mean difference (MD) OR weight difference (WMD) were used as effect analysis for continuous variables. Heterogeneity was determined by χ2 test and I^2^ test. If there was no clinical heterogeneity (P > 0.1, I^2^<50%), fixed effects model (FEM) was used for meta-analysis. If clinical heterogeneity exists (I^2^> 50%), the random-effects model (REM) was used for meta-analysis and the source of heterogeneity was discussed. If necessary, subgroup analysis was performed. If the source of heterogeneity could not be determined, case by case elimination was used to investigate the source of heterogeneity. As less than 10 outcome indicators were included in each group, no funnel plot was made.

## Results

### Literature screening results

**Fig 1:**
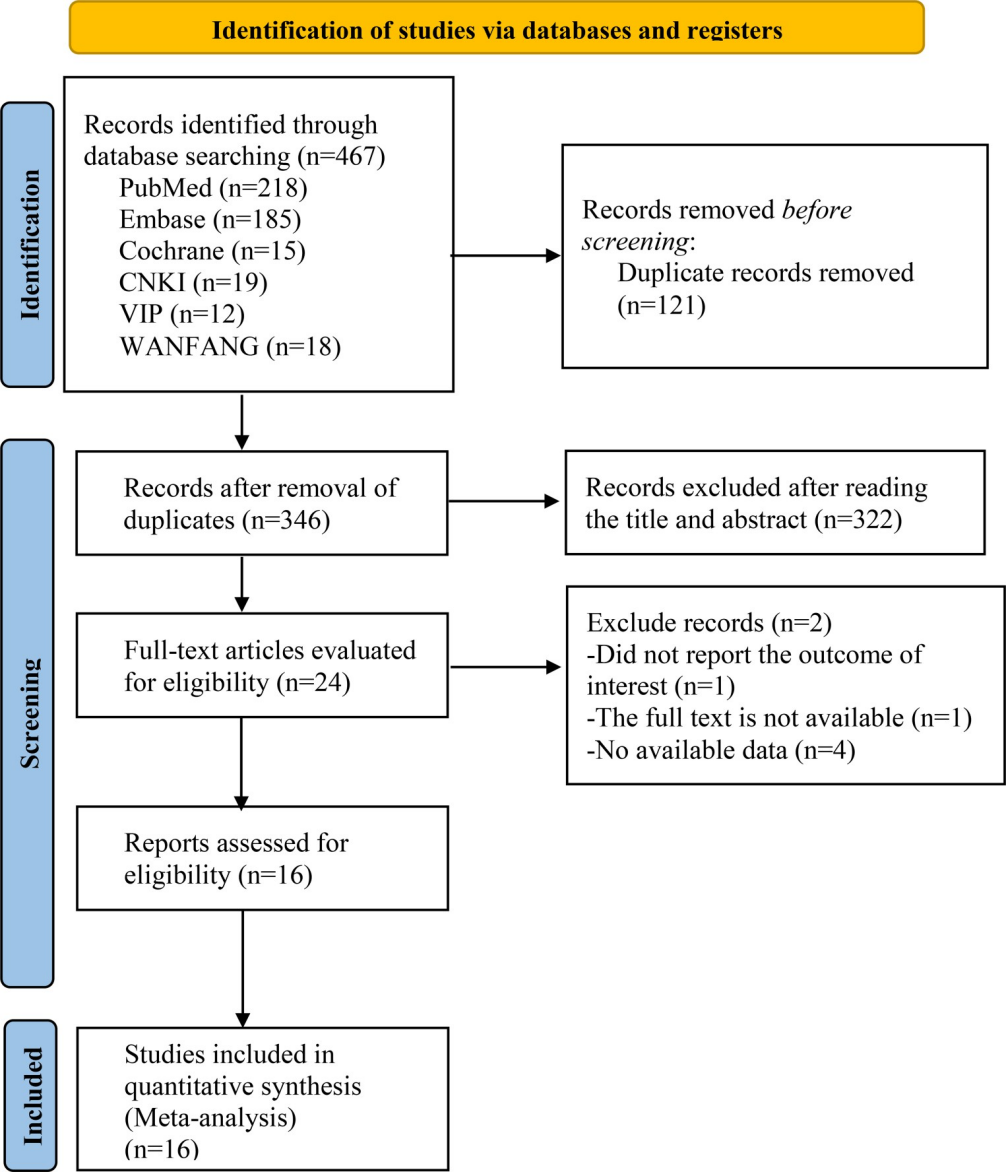
Literature search process.

The flow chart of literature selection is shown in Fig 1. After selection, 16 articles were finally included in the quantitative analysis, and a total of 1782 people participated. The main characteristics, interventions, and outcome measures of the selected literature are shown in Table 1.

**Table 1:** Outcome indicators.

Study	Study design	Age(years) (E/C)	Sample size (E/C)	Interventions (E/C)	Follow-up (M)	Outcome indicators
LUO YL 2015 [11]	RCT	80.7±7.8/80±7.4	35/37	MT+CR/MT	3–12	LVEF;LVDD;MLHFQ;BNP;NYHA;
XIAO YL 2021 [12]	RCT	66.78±4.43/65.27±4.37	35/35	MT+CR/MT	3	Total effective rate of nursing; 6MWT;LVEF;LVDD; LVDs
SHEN F 2019 [18]	RCT	67.5±11.5/68.4±10.3	50/50	MT+CR/MT	3	6MWT;PRO-BNP
LIU LP 2020 [19]	RCT	75.21±2.22/75.79±2.11	25/25	MT+CR/MT	6	MLHFQ;ESES;6MWT;PRO-BNP
LIAO JX 2020 [13]	RCT	67.42±5.63/66.50±5.74	52/52	MT+CR/MT	3	MLHFQ;CPET;Borg;6MWT;PRO-BNP;LVEF;Readmission rate HPLP-II
KANG TD 2017 [14]	RCT	69±7/68±7	44/44	MT+CR/MT	6	LVEF;LVDD;LVDs;MLHFQ;6MWT;PRO-BNP;CTNI; Readmission rate;Death rate
JIN LJ 2019 [20]	RCT	70.03±6.71/69.92±6.63	35/35	MT+CR/MT	3	6MWT;MLHFQ;Readmission rate
HUI HP 2020 [21]	RCT	87.3±6.4/86.3±7.8	58/54	MT+CR/MT	3	6MWT;peakSV;peak CO;TPVR;SF-36
HU YH 2020 [15]	RCT	62.53±8.75/63.05±8.54	47/47	MT+CR/MT	3	LVEF;LVDD;LVDs
FANG KH 2020 [20]	RCT	68.29±4.07/68.56±4.22	75/75	MT+CR/MT	6	LVEF;LVDD;LVDs;PRO-BNP;CTNI;SDS;SAS;MLHFQ
DING F 2020 [24]	RCT	68.92±4.62/68.47±4.42	38/38	MT+CR/MT	3	CD-RISC;MLHFQ;6MWT
ZU LFYYKM 2018 [17]	RCT	68.1±6.3/67.5±5.8	38/37	MT+CR/MT	6	6MWT;LVEF;LVDD; BNP
Austin 2005 [23]	RCT	71.9±6.3/71.8±6.8	100/100	MT+CR/MT	2–6	6MWT;MLHF;QOL;NYHA;Borg;Readmission rate; Death rate
Austin 2008 [25]	RCT	71.9/71.8	100/100	MT+CR/MT	6–60	6MWT;MLHF;QOL; Borg;
Dalal 2019 [26]	RCT	69.7±10.9/69.9±11	107/109	MT+CR/MT	4–12	QOL;MLHFQ;HADS;SCHFI; ISWT
Davidson 2010 [22]	RCT	71.6/73.9	53/52	MT+CR/MT	3–12	MLHFQ;HFNAQ;6MWT;NYHA;

RCT: Randomized controlled trial E: Experimental group C: Control group MT: Medication Therapy CR: CR M: Month LVEF: Left ventricular ejection fraction LVDD: Left ventricular end-diastolic diameter MLHFQ: Minnesota Living with Heart Failure Questionnaire BNP:B natriuretic peptide PRO-BNP: PRO-B natriuretic peptide 6MWT: 6-min walk test LVDs: Left ventricular end-diastolic diameter QOL: Quality of life scale ESES: Exercise self-efficacy Scale CPET: Cardiopulmonary exercise test Borg: Dyspnea score HPLP-II: Health promotes lifestyle peakSV: Peak stroke volume peakCO: Peak Cardiac Output TPVR: Total peripheral vascular resistance SF-36: Short Form-36 Health Status Questionnaire SDS: Self-Rating Depression Scale SAS: Self-rating anxiety scale CD-RISC: CD-Resilience Scale HFNAQ: Heart failure needs Assessment questionnaire SCHFI: Heart failure Self-care Scale ISWT: Incremental shuttle walking test.

### Results of risk of bias assessment

**Fig 2:**
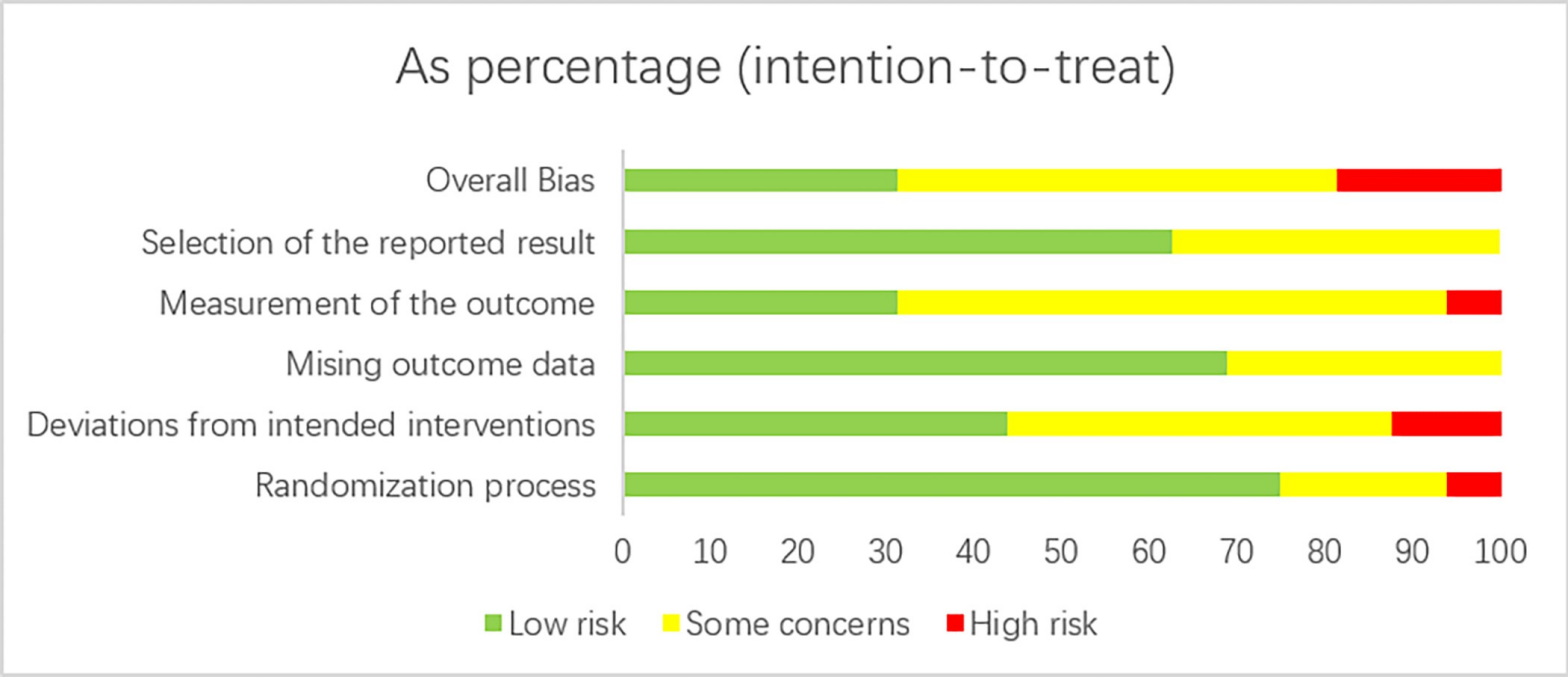
Risk of bias in the literature.

**Fig 3:**
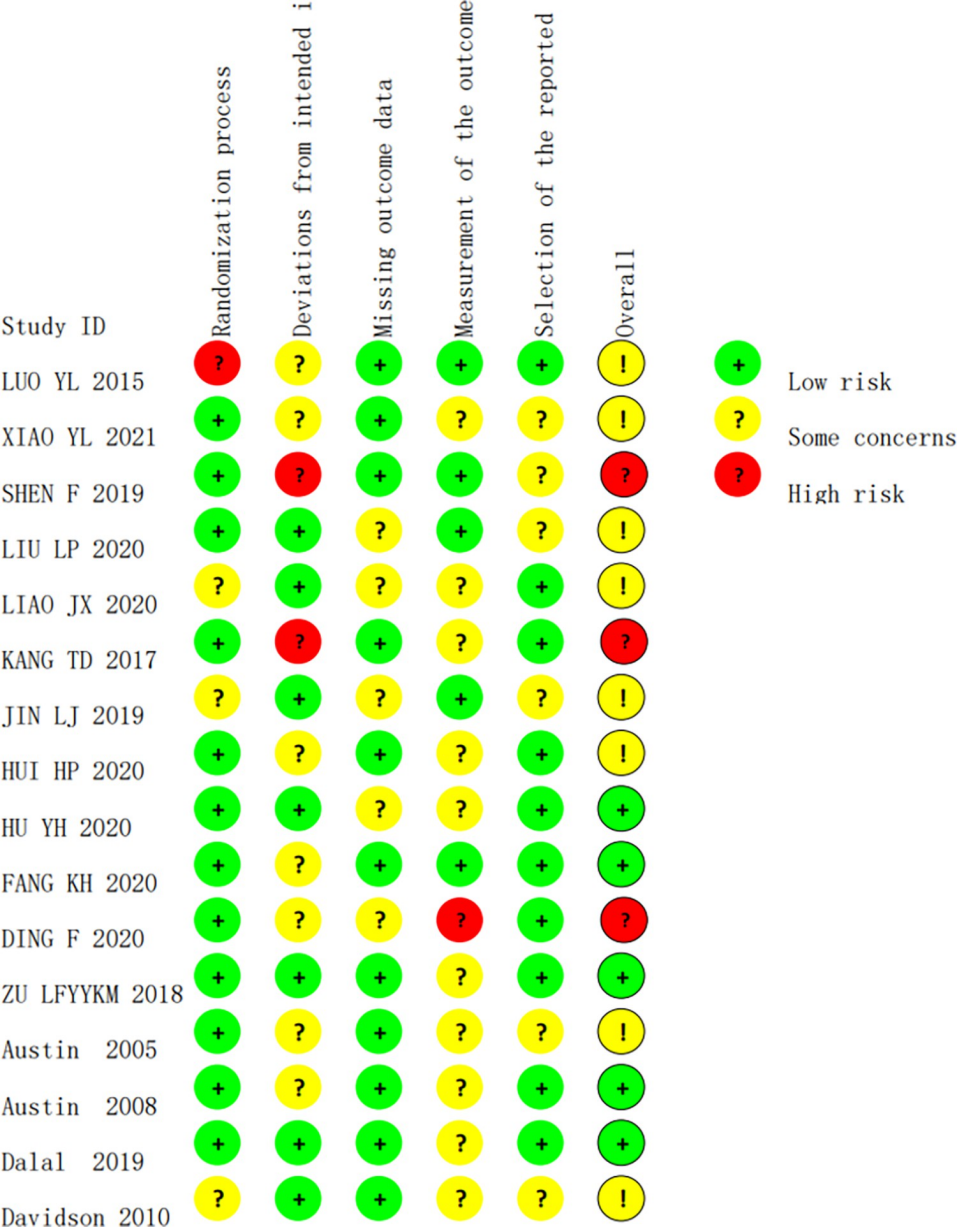
Literature bias risk.

Risk of bias was assessed in 16 studies. The specific evaluation and risk bias assessment are shown in Figs 2 and 3.

### GRADE evidence rating results

The results of GRADE evidence are shown in S1 Fig.

### Meta-analysis results

#### Influence of CR on LVEF of patients

**Fig 4:**
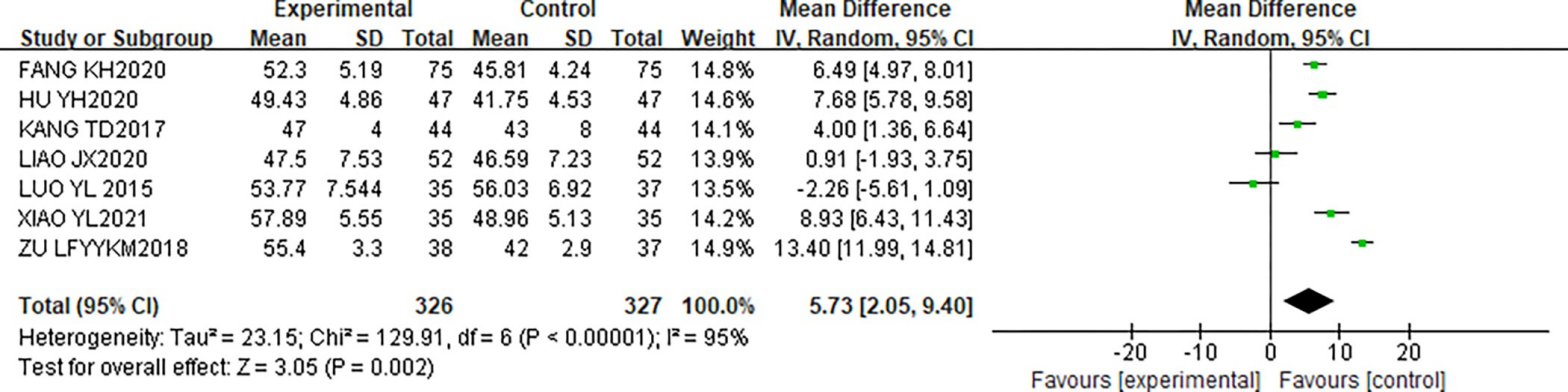
Influence of CR on LVEF of patients.

This study included 7 articles [11–17] reporting the effect of CR on LVEF in patients. LVEF was detected in 327 patients in the experimental group and 326 patients in the control group. After the heterogeneity test, the results showed that I^2^ = 95%, P < 0.00001, indicating that there is heterogeneity in the literature, and REM was used for analysis. The results of meta-analysis showed that [MD = 5.73, 95%CI(2.05,9.40), P = 0.002]. Therefore, there was a statistically significant difference in LVEF between the two groups after CR (P<0.05). The forest map is shown in Fig 4.

#### Influence of CR on LVEDD of patients

**Fig 5:**
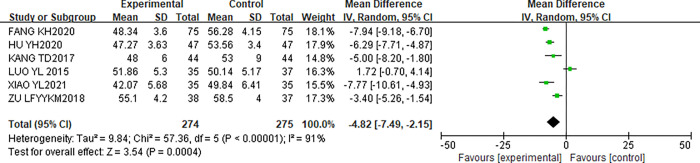
Influence of CR on LVEDD of patients.

This study included 6 articles [11,12,14–17] reporting the effect of CR on LVDD in patients. LVDD was detected in 274 patients in the experimental group and 275 patients in the control group. After the heterogeneity test, the results showed that I^2^ = 91%, P<0.00001, indicating that there is obvious heterogeneity in the literature. Analysis was performed using REM. The results of the meta-analysis showed that [MD = -4.82,95%CI(-7.49,-2.15), P = 0.0004]. Therefore, there was a statistically significant difference in LVDD between the two groups after CR (P<0.05). The forest map is shown in Fig 5.

#### Influence of CR on 6MWT of patients

**Fig 6:**
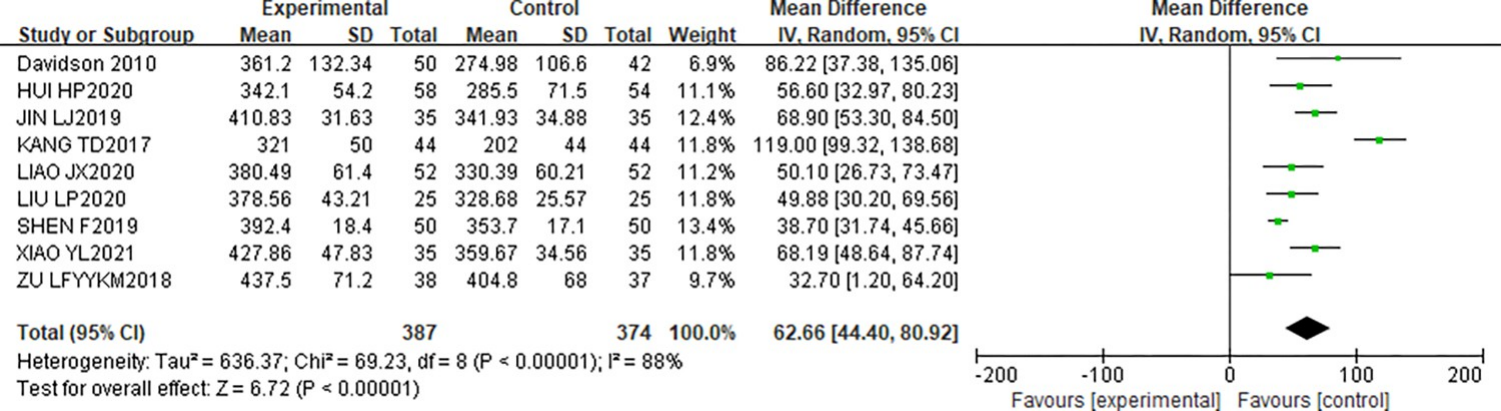
Influence of CR on 6MWT of patients.

This study included 9 articles [12–14,17–22] reporting the effect of CR on 6MWT in patients. 6MWT was detected in 387 patients in the experimental group and 374 patients in the control group. After the heterogeneity test, the results showed that I^2^ = 88%, P<0.00001, indicating that there is obvious heterogeneity in the literature. Analysis was performed using REM. The results of meta-analysis showed that [MD = 62.66,95%CI(44.40,80.92), P<0.00001]. Therefore, there was a statistically significant difference in 6MWT between the two groups after CR (P<0.05). The forest map is shown in Fig 6.

#### Influence of CR on readmission rate of patients

**Fig 7:**
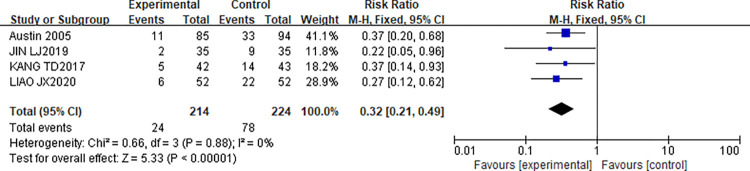
Influence of CR on Readmission rate of patients.

This study included 4 articles [13,14,20,23] reporting the effect of CR on Readmission rate in patients. The readmission rate was calculated for 214 patients in the experimental group and 224 patients in the control group. After the heterogeneity test, the results showed that I^2^ = 0%, P = 0.88, indicating that there is no heterogeneity in the literature. Analysis was performed using FE. The results of the meta-analysis showed that [OR = 0.32,95%CI(0.21,0.49), P<0.000001]. Therefore, there was a statistically significant difference in the rate of readmission after CR between the two groups (P<0.05). The forest map is shown in Fig 7.

## Discussion

### Methodological quality evaluation of the included literature

A total of 16 RCTs [11–26] were selected for this meta-analysis, of which 12 were randomized, 3 were randomized concealment, and 3 were blinded to outcome assessors. The poor quality of some included literatures may lead to a higher risk of bias, thus affecting the accuracy of the system evaluation. All studies were compared with baseline data and showed comparability.

### Heterogeneity analysis of this study

In this meta-analysis, there was heterogeneity in the three outcome index groups of LVEF, LVEDD and 6MWT. First, we checked the original data and inclusion methods of the included study and found no errors. Then, the combined mean of the remaining studies remained stable by case elimination method. Finally, we performed subgroup analysis and found no difference from the overall analysis. The main sources of heterogeneity were as follows: 1. Most selected RCTS were of low quality and poor methodological quality; 2. Assessment methods are different for each study; 3. There are differences in nationality, living habits, underlying diseases and follow-up time of included samples; 4. Differences exist in the level of researchers and intervention methods.

### Primary outcomes

CHF, also known as a malignant disease in heart disease [27], is a clinical syndrome characterized by impaired ventricular filling or ejection capacity due to structural abnormalities of the heart or cardiac dysfunction and it is the ultimate destination for various heart diseases. The 2018 American Heart Association (AHA) report stated that there are currently about 6.5 million CHF patients in the United States, and the prevalence of CHF in the elderly will increase by 46% by 2030 [28]. According to the "China Cardiovascular Disease Report 2017" (6), there are 4.5 million heart failure patients in my country, and the hospitalized heart failure mortality rate is 5.3%, and the incidence of CHF in the elderly will continue to rise in the future. In recent years, the treatment and management of CHF has progressed rapidly thanks to the development of drug and non-drug treatment options. However, most patients with CHF still face the situation of poor quality of life, repeated disease and low survival rate. It is currently believed that the treatment goals of CHF control symptoms, improve quality of life, inhibit myocardial remodeling, delay disease progression, and prevent disease recurrence, thereby reducing heart failure hospitalization and mortality. Compared with heart failure patients of other ages, elderly patients with heart failure have the characteristics of weakened tissues and organs, reduced exercise tolerance, more co-morbidities, poor compliance, high stress, and pessimism [29]. In addition to conventional treatment, effective CR is particularly important. important. CR can slow down and inhibit the progression of atherosclerosis through the comprehensive intervention of five prescriptions: drug, exercise, nutrition, psychological improve and patient education; improve exercise tolerance, quality of life; the function of circulatory system and reduce cardiovascular disease Mortality, control of risk factors, and overall improvement of patient outcomes. CR for CHF began at the end of the last century. Evidence-based medical evidence confirms that CR can reduce the rehospitalization rate and mortality of patients with CHF, improve exercise tolerance and quality of life, and control social medical care. This study systematically evaluated the clinical efficacy of CR on elderly patients with CHF, in order to provide a reference for clinical diagnosis and treatment. Echocardiography is a common method for evaluating cardiac function, with a positive diagnostic rate of 95.48% [30]. LVEF and LVEDD are commonly used parameters of cardiac ultrasound, and LVEF value is positively correlated with myocardial contractility; LVEDD can reflect the degree of coronary atherosclerosis in patients to a certain extent [31]. Among the 16 included studies, 7 studies [11–17] and 6 studies [11,12,14–17] performed LVEF, LVEDD detection. The analysis showed that compared with conventional treatment, CR for elderly patients with CHF can significantly improve LVEF, reduce LVEDD, and improve cardiac function. 6MWT is a sensitive indicator for evaluating exercise tolerance and functional compensation in patients with heart failure. It is non-invasive, simple, safe, and highly reproducible. It is positively correlated with exercise tolerance and cardiac function [32]. Of the 16 included studies, nine studies [12–14,17–22] assessed changes in 6MWT after participation in CR in patients with heart failure. The results show that compared with conventional treatment, CR can significantly increase the 6MWT distance and improve exercise tolerance in elderly patients with CHF. The readmission rate is a huge challenge faced by heart failure patients, an important indicator for evaluating the patient’s cardiac function and prognosis, and a key factor related to medical costs. Of the 16 included studies, 4 studies [13,14,20,23] measured changes in readmission rates in patients with heart failure who participated in cardiac rehabilitation. Results showed that performing CR significantly reduced readmission rates in elderly patients with CHF compared with usual care.

### Study limitations and future recommendations

This systematic analysis has the following limitations: (1) Jadad quality rating scale was used to evaluate, and all 16 included studies were randomized, and 3 studies [23,25,26] had incomplete descriptions of randomization; 3 studies [22,23,25] reported allocation concealment, and 3 studies [22,23,25] had Patients were blinded; most of the samples were small in size and low in quality; (2) most of the studies were combined with obvious heterogeneity, which was considered to be related to the study design, study methodology, sample differences, etc.; (3) none of the included literature found Negative results may have publication bias; (4) The duration of CR is very different and carries a potential risk of bias; (5) the included literatures are mostly domestic studies.

Based on the above limitations, the following suggestions are put forward for future research: (1) It is necessary to carry out multi-center, large-sample, high-quality double-blind randomized controlled studies; (2) To develop a more scientific and reasonable research design, high-quality methodological research, pay attention to The application of randomization, allocation concealment and blinding (3) multi-regional and multi-ethnic research needs to be carried out; (4) appropriately increase the follow-up time.

## Conclusions

In this study, 16 articles were included in a meta-analysis to discuss the impact of CR on elderly patients with CHF. Significant differences were found between the two groups in terms of LVEF, LVEDD, readmission rate, and 6MWT (P < 0.05). CR can significantly improve cardiac function, increase exercise tolerance, reduce the degree of arteriosclerosis, improve patient prognosis, and reduce readmission rates in elderly patients with heart failure.

## Supporting information

S1 Checklist PRISMA 2020 checklist. (PDF)

S1 Fig GRADE evidence rating results. (TIF)

## References

[1] Hua Wang YL. Chinese Heart Failure Diagnosis and Treatment Guidelines 2018. 2018;46(10):760–89. 10.3760/cma.j.issn.0253-3758.2018.10.004 30369168

[2] Ventura HO, Silver MA. Observations and Reflections on the Burden of Hospitalizations for Heart Failure. Mayo Clinic proceedings. 2017;92(2):175–8. doi: 10.1016/j.mayocp.2016.12.009 28160868

[3] Driscoll A, Worrall-Carter L, Stewart S. Rationale and design of the National Benchmarking and Evidence-based National Clinical Guidelines for CHF Management Programs Study. The Journal of cardiovascular nursing. 2006;21(4):276–82. 16823280 10.1097/00005082-200607000-00007

[4] Capriotti T, Micari M. CHF Treatment With the Left Ventricular Assist Device. Home healthcare now. 2019;37(4):190–7. 31274581 10.1097/NHH.0000000000000777

[5] Dokainish H, Teo K, Zhu J, Roy A, AlHabib KF, ElSayed A, et al. Global mortality variations in patients with heart failure: results from the International Congestive Heart Failure (INTER-CHF) prospective cohort study. The Lancet Global health. 2017;5(7):e665–e72. doi: 10.1016/S2214-109X(17)30196-1 28476564

[6] Weiwei Chen RG, Lisheng Liu, Manlu Zhu, Wen Wang, Yongjun Wang, Zhaosu Wu, et al. China Cardiovascular Disease Report 2017. Chinese Circulation Journal. 2018;33(01):1–8. 10.1093/eurheartj/suw030 28533724

[7] Williamson T, Moran C, Chirico D, et al. Cancer and cardiovascular disease: The impact of cardiac rehabilitation and cardiorespiratory fitness on survival. Int J Cardiol. 2021 Nov 15;343:139–145. doi: 10.1016/j.ijcard.2021.09.004 34506825 PMC9178663

[8] Lavie CJ, Arena R, Swift DL, Johannsen NM, Sui X, Lee DC, et al. Exercise and the cardiovascular system: clinical science and cardiovascular outcomes. Circulation research. 2015;117(2):207–19. doi: 10.1161/CIRCRESAHA.117.305205 26139859 PMC4493772

[9] de Gregorio C. Physical Training and CR in Heart Failure Patients. Advances in experimental medicine and biology. 2018;1067:161–81. 29453669 10.1007/5584_2018_144

[10] Moher D, Liberati A, Tetzlaff J, Altman DG. Preferred reporting items for systematic reviews and meta-analyses: the PRISMA statement. International journal of surgery (London, England). 2010;8(5):336–41. doi: 10.1016/j.ijsu.2010.02.007 20171303

[11] Luo YL. Effects of CR on cardiac function and quality of life in elderly patients with CHF. 2015:493–4.

[12] Yanlan Xiao LX, Min Yang. Application effect of exercise-based CR nursing in elderly patients with CHF. China Modern Medicine. 2021;28(26):240–3.

[13] Liao JX. Influence of comprehensive intervention of cardiopulmonary rehabilitation based on health promotion model on elderly patients with CHF. University of South China. 2020.

[14] Kang T.D. Application of CR exercise in the treatment of elderly patients with CHF Chinese Medicine. 2017;12(2):171–4.

[15] Yihua Hu LL, Rui Jin. Effects of CR exercise therapy on CHF with coronary heart disease in the elderly. Electronic Journal of Clinical Medicine. 2020;7(18).

[16] Kehua Fang FY. Effects of CR exercise on cardiac function and adverse psychological mood in elderly patients with CHF. Clinical Medicine. 2020;40(09).

[17] LFYYKM ZU. Observation on the efficacy of CR exercise in the treatment of elderly CHF. China Health Nutrition. 2018;28(17).

[18] Shen YX F., Li W. Observation on the effect of CR exercise therapy on CHF with coronary heart disease in the elderly. Massage and Rehabilitation Medicine. 2019;10(08):11–2.

[19] Liping Liu Tl Quanlin Zou. Clinical effect of CR on the safety of elderly patients with CHF. Chinese Journal of Clinical Rational Drug Use. 2020;13(25).

[20] JIN LJ. Interpretation of the effect of CR exercise therapy on CHF with coronary heart disease in the elderly. Diet and Health Care. 2019;6(35).

[21] Haipeng Hui LZ, Xuan Zhou, Yuan Liu, Dawei Yin, Meng Gao, Yongmei Hu, et al. Clinical efficacy of cardiopulmonary integrated CR in elderly patients with CHF China Cardiovascular Research. 2020;18(10):880–3.

[22] Davidson PM, Cockburn J, Newton PJ, Webster JK, Betihavas V, Howes L, et al. Can a heart failure-specific CR program decrease hospitalizations and improve outcomes in high-risk patients? European journal of cardiovascular prevention and rehabilitation: official journal of the European Society of Cardiology, Working Groups on Epidemiology & Prevention and CR and Exercise Physiology. 2010;17(4):393–402. 10.1097/HJR.0b013e328334ea56 20498608

[23] Austin J, Williams R, Ross L, Moseley L, Hutchison S. Randomised controlled trial of CR in elderly patients with heart failure. European journal of heart failure. 2005;7(3):411–7. 15718182 10.1016/j.ejheart.2004.10.004

[24] Fei Ding ZC. Effects of group positive psychological intervention combined with standardized first-stage exercise rehabilitation on resilience and quality of life in elderly patients with CHF. Clinical Medicine Research and Practice. 2020;5(25).

[25] Austin J, Williams WR, Ross L, Hutchison S. Five-year follow-up findings from a randomized controlled trial of CR for heart failure. European journal of cardiovascular prevention and rehabilitation: official journal of the European Society of Cardiology, Working Groups on Epidemiology & Prevention and CR and Exercise Physiology. 2008;15(2):162–7. 10.1097/HJR.0b013e3282f10e87 18391642

[26] Dalal HM, Taylor RS, Jolly K, Davis RC, Doherty P, Miles J, et al. The effects and costs of home-based rehabilitation for heart failure with reduced ejection fraction: The REACH-HF multicentre randomized controlled trial. European journal of preventive cardiology. 2019;26(3):262–72. doi: 10.1177/2047487318806358 30304644 PMC6376602

[27] Stewart S, MacIntyre K, Hole DJ, Capewell S, McMurray JJ. More ’malignant’ than cancer? Five-year survival following a first admission for heart failure. European journal of heart failure. 2001;3(3):315–22. doi: 10.1016/s1388-9842(00)00141-0 11378002

[28] Benjamin EJ, Virani SS, Callaway CW, Chamberlain AM, Chang AR, Cheng S, et al. Heart Disease and Stroke Statistics-2018 Update: A Report From the American Heart Association. Circulation. 2018;137(12):e67–e492. doi: 10.1161/CIR.0000000000000558 29386200

[29] Xiaohua Shen HL. Investigation on healthy lifestyle of the elderly. Zhejiang Preventive Medicine. 2013;25(01):31–2.

[30] Frolov DS, Salukhov V, Shustov SB, Lokshina TR, Mechnikov VEJHoN-WSMUnaII. Features of the structural and functional condition of the myocardium in patients with chronic kidney disease and CHF. 2019;11(3):79–84.

[31] Cameli M, Sciaccaluga C, Loiacono F, Simova I, Miglioranza MH, Nistor D, et al. The analysis of left atrial function predicts the severity of functional impairment in CHF: The FLASH multicenter study. International journal of cardiology. 2019;286:87–91. 30955880 10.1016/j.ijcard.2019.03.063

[32] Yulong Wang CX, Xiaoli Hu, Zhihua Lu, Ye Tang, Lili Huang. The value of 6-min walking test combined with plasma N-terminal precursor brain natriuretic peptide in evaluating the prognosis of patients with CHF. Chinese journal of gerontology. 2020;39(12):1411–4.

