# Supplementary figures and images for: Effects of cardiac rehabilitation on elderly patients with Chronic heart failure: A meta-analysis and systematic review
Source: PLoS One. 2022 Aug 25;17(8):e0273251. doi: 10.1371/journal.pone.0273251 (PMC9409506; doi:10.1371/journal.pone.0273251)

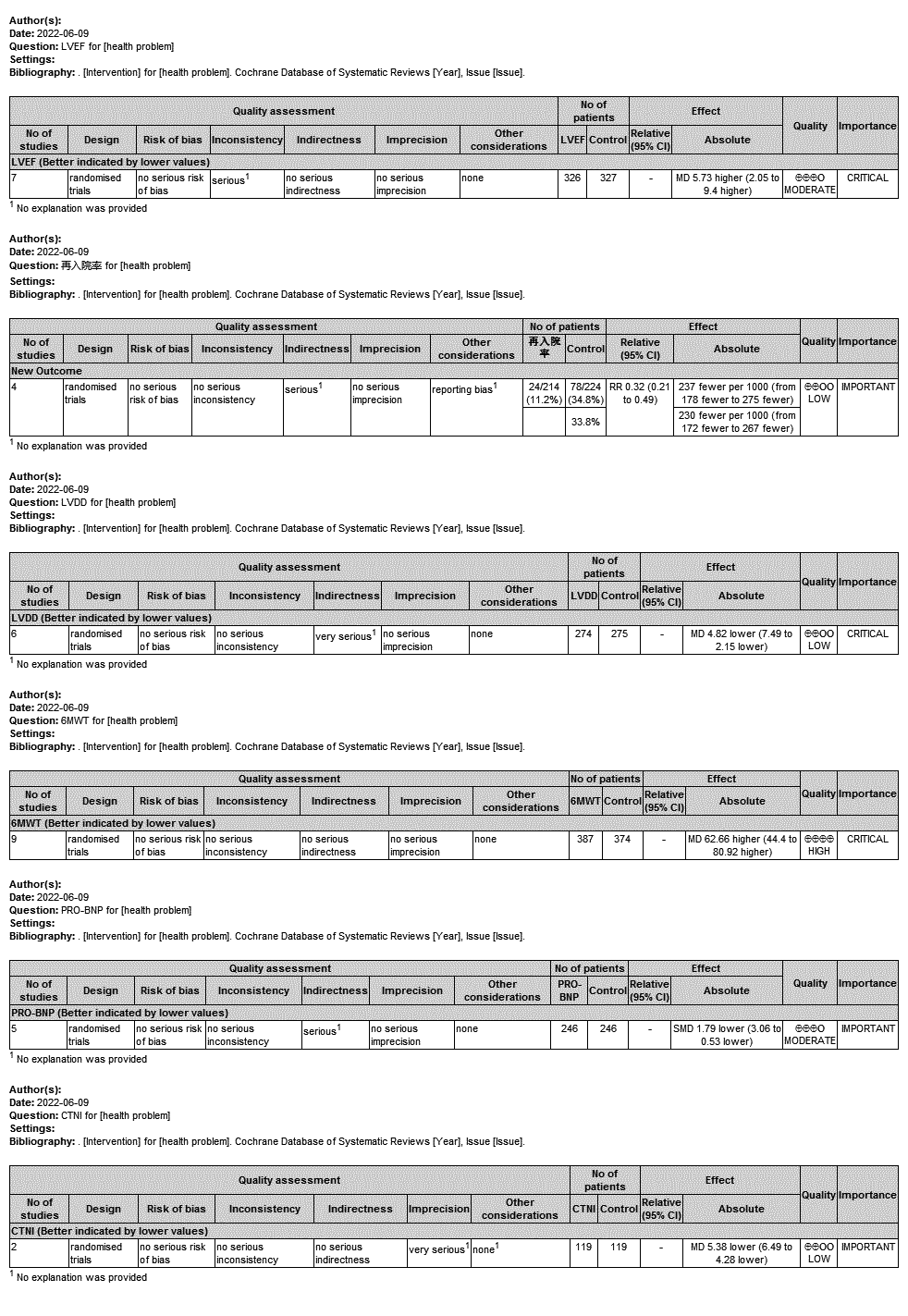

Supplement: S1 Fig — (TIF) [file pone.0273251.s002.tif]
